# *Solanum hydroides* (Solanaceae): a prickly novelty from the land of the sugar loaves, central Brazilian Atlantic Forest

**DOI:** 10.3897/phytokeys.139.46635

**Published:** 2020-02-03

**Authors:** Yuri Fernandes Gouvêa, Luiza Fonseca Amorim de Paula, João Renato Stehmann, Leandro Lacerda Giacomin

**Affiliations:** 1 Departamento de Botânica, Instituto de Ciências Biológicas, Universidade Federal de Minas Gerais – UFMG, Av. Antônio Carlos, 6627, Pampulha, Belo Horizonte, CEP 31270-901, MG, Brazil; 2 Departamento de Genética, Ecologia e Evolução, Instituto de Ciências Biológicas, Universidade Federal de Minas Gerais – UFMG, Av. Antônio Carlos, 6627, Pampulha, Belo Horizonte, CEP 31270-901, MG, Brazil; 3 Instituto de Ciências e Tecnologia das Águas & Herbário HSTM, Universidade Federal do Oeste do Pará, Av. Mendonça Furtado, 2946, Santarém, CEP 68040-050, PA, Brazil

**Keywords:** Leptostemonum clade, Brazil, inselbergs, new species, eglandular trichomes, Clado Leptostemonum, Brasil, inselbergs, espécie nova, tricomas eglandulares

## Abstract

*Solanum hydroides* Gouvêa & Giacomin, **sp. nov.**, is described from central Brazilian Atlantic Forest. It is known from only three localities in Espírito Santo and Minas Gerais states, where granitic/gneissic outcrops (inselbergs or sugar loaves) are ubiquitous. The new species, here described, belongs to Solanum subgenus Leptostemonum (or the Leptostemonum clade; i.e. the spiny solanums) and is morphologically related to *S. hexandrum* Vell. and *S. sublentum* Hiern, with which it shares the shrubby habit, decurrent leaf bases and well-developed calyces that become accrescent, covering glabrous fruits. *Solanum hydroides* is unique in its combination of comparatively more delicate habit, indumentum of exclusively stellate eglandular trichomes, accrescent but never inflated fruiting calyces that only partially cover the fruits and comparatively shortly lobed and strictly white corollas. The species is threatened with extinction and assessed as Vulnerable (VU), based on the IUCN criteria.

## Abstract (pt)

*Solanum hydroides* Gouvêa & Giacomin, sp. nov., é aqui descrita para região central da Floresta Atlântica brasileira. Ela é conhecida apenas para três localidades, nos estados do Espírito Santo e Minas Gerais, em uma região onde afloramentos graníticos/gnáissicos (inselbergues ou pães de açúcar) são onipresentes. A nova espécie pertence a Solanum subgen. Leptostemonum (ou *Solanum* clado Leptostemonum, i.e. espécies espinhentas com tricomas estrelados) e é morfologicamente semelhante a *S. hexandrum* Vell. e *S. sublentum* Hiern, com quem compartilha o hábito arbustivo, a base das lâminas foliares decurrentes e cálices bem desenvolvidos, acrescentes em seus frutos glabros. *Solanum hydroides* é, contudo, a única espécie que apresenta a seguinte combinação de caracteres: hábito delicado, indumento composto apenas por tricomas estrelados egandulares, cálices frutíferos acrescentes, mas nunca inflados, que cobrem parcialmente os frutos e corolas com lóbulos curtos e estritamente brancas. A espécie é considerada como ameaçada de extinção, na categoria Vulnerável (VU), segundo os critérios da IUCN.

## Introduction

*Solanum* L. (Solanaceae) is one of the largest genera of flowering plants with about 1,400 species, mostly distributed in the Neotropics (Nee 1999; Frodin 2004; Weese and Bohs 2007). Some species have great economic importance, being used as food, such as the potato (*S. tuberosum* L.), tomato (*S. lycopersicum* L.) and eggplant (*S. melongena* L.) and are cultivated practically all over the world; other species have medicinal and ornamental uses (Hawkes 1999). Several species behave as pioneers and are therefore fundamental in the ecological succession, especially in tropical forests (Guariguata and Ostertag 2001). In Brazil, the Atlantic Forest stands out as a centre of diversity for the genus, with 184 species recorded thus far, of which 98 are endemic (Stehmann et al. 2009; Flora do Brasil 2020 under construction 2019). In addition to species level endemism, the Atlantic Forest domain harbours several endemic lineages within *Solanum*, such as the Asterophorum and Inornatum species groups (Giacomin and Stehmann 2014; Gouvêa and Stehmann 2019). In fact, the domain shows an ascending curve of newly described taxa in the last years, demonstrating the insufficiency of taxonomic knowledge (Linnaean shortfall) (Sobral and Stehmann 2009).

The Atlantic Forest domain is considered one of the 36 hotspots of global biodiversity, defined as a region of the world with a large number of endemics and highly endangered, with its original coverage extremely reduced (Myers et al. 2000; Mittermeier et al. 2004, https://www.conservation.org/priorities/biodiversity-hotspots). It comprises an extensive predominantly forest belt, of more than 1.2 million square kilometers, occurring along coastal areas from Rio Grande do Norte to Rio Grande do Sul states in Brazil, with continental intrusions reaching Argentina and Paraguay (Stehmann et al. 2009). Biogeographically, three regions (North, Central and South Atlantic Forest) with distinct paleoecological and floristic histories have been recognised along this longitudinal distribution (Carnaval and Moritz 2008; Carnaval et al. 2014).

The Central Atlantic Forest is delimited to the south by the Doce River and to the north by the São Francisco (Carnaval et al. 2014). Compared with the other two regions, it presented greater paleoclimatic stability during the Pleistocene climatic cycles (Carnaval and Moritz 2008; Silveira et al. 2019). In South-eastern Brazil, the area corresponding to the Doce, Mucuri and Jequitinhonha basins in Minas Gerais and Espirito Santo states, is amongst the most deforested regions of the domain and huge gaps in knowledge of its biodiversity still exist (Oliveira-Filho et al. 2005; Martinelli 2007; Oliveira et al. 2016). From a landscape perspective, the region is distinctive in its large concentration of granitic/gneissic rock outcrops, also known as inselbergs, with a so-called sugar loaf morphology (Ab'Sáber 1967) and, thus, the area was christened as the land of the sugar loaves (Sugar Loaf Land; de Paula et al. 2016). Inselbergs of this region in general, with their steep slopes, are inhabited by patches of herbaceous-shrubby vegetation, mostly stress-tolerant species (de Paula et al. 2015) and are usually surrounded by forest remnants of varying sizes (de Paula et al. 2017). These outcrops, which stand out in the landscape as islands, provide a broad ecotonal range and islands of open habitat, which favour the occurrence of woody species that demand light (pioneers), including many species of *Solanum* (Guariguata and Ostertag 2001).

During a floristic inventory of one inselberg located in the Brazil´s South-Eastern region (de Paula et al. 2017) and after more extensive herbaria revision by the authors, an undescribed *Solanum* species was revealed. It belongs to the group that comprises prickly plants with stellate trichomes [Solanum subg. Leptostemonum Bitter or the Leptostemonum Clade] and its discovery supports the need for inventories in the region. We describe here this new taxon with illustrations, comments on habitat, geographic distribution, ecology and a preliminary conservation status.

## Material and methods

Observations are all based on examination of herbarium specimens from BHCB, CEPEC, HUEFS, MBML, RB and UEC (acronyms follow Index Herbariorum; http://sweetgum.nybg.org/science/ih/), as well as in-field observations by LFAP and YFG. Type specimens of related taxa were consulted in BR, G and M or through high resolution images available on the Global Plants website (https://plants.jstor.org) and original descriptions of related species were checked when necessary. Measurements of reproductive characters comprise the dimensions of both fresh and dried materials. The terms used to describe the morphological character states are based on Radford et al. (1976). We assessed the conservation status of *S. hydroides* using IUCN Red List Categories and Criteria (IUCN 2019) with extent of occurrence (EOO) and area of occupancy (AOO) measured using GeoCat (Bachman et al. 2011; http://geocat.kew.org/). For the AOO estimation, a cell size of 4 km^2^ was used. A full dataset with the examined material of *S. hydroides* is given as a supplementary file (Suppl. material 1). The distribution of the most morphologically similar species (namely *Solanum hexandrum* Vell. and *S. sublentum* Hiern.) were mapped together with the known records of *S. hydroides* and the full dataset used for these species is also given as supplementary material (Suppl. material 2), which was downloaded from the speciesLink platform (http://www.splink.org.br/) and properly cleaned prior to mapping. For SEM seed observations, seeds of ripe fruits were obtained from dried material, mounted on aluminium stubs, coated with gold-palladium in a Hummer 6.2 sputtering system (Anatech, Union City, CA, U.S.A) and observed with a JEOL JSM-5410LV SEM (JEOL, Tokyo, Japan) at 10 kV at the Pfizer Plant Research Laboratory at The New York Botanical Garden.

## Taxonomic treatment

### Solanum hydroides

Taxon classificationPlantae

Gouvêa & Giacomin sp. nov.

8AB36703-BB11-5F79-8622-5AA6FB98E31E

urn:lsid:ipni.org:names:77204874-1

Figure 1

#### Diagnosis.

Differs from *S. sublentum* Hiern in its indumentum of strictly stellate eglandular trichomes and in its accrescent, but not inflated, cupuliform fruiting calyx; also differs from *S. hexandrum* Vell. in its more delicate habit, smaller flowers with white shallowly stellate corollas and in having accrescent, but not inflated, fruiting calyces that partially cover the mature fruits.

#### Type.

BRAZIL. Minas Gerais: Mun. Teófilo Otoni, afloramento rochoso lado esquerdo da MG-418, cerca de 30 km norte de Teófilo Otoni, 17°51'22"S, 41°15'39"W, 560 m elev., 27 Jan 2014 (fl, fr), *L.F.A. de Paula*, *L. Azevedo, R. Fernandes & J. R. Stehmann 669* (holotype: BHCB [BHCB053358]; isotype: RB, to be distributed).

#### Description.

Shrubs 1–1.5 m tall, erect, armed. Branches directed upwards and spreading. Young stems moderately pubescent to tomentose and sparsely to moderately prickly; pubescence of ochraceo-ferruginous to purple-tinged porrect short- to long-stalked stellate trichomes, with multiseriate 0.5–1.5 mm long stalks, the rays 4-8, 0.5–1 mm long, the midpoints 1– to 2–celled, always shorter than the rays; prickles 4–6 mm long, 2–6 mm wide at the base, broad-based and recurved. Bark of older stems glabrescent, drying olivaceous to brown. Sympodial units plurifoliate, the leaves not geminate. Leaves simple, nearly entire to shallowly lobed, the blades 2.8–12.1(21.8) cm long, 2.2–7.5(10.1) cm wide, elliptic to ovate, membranous, slightly discolorous when dry; adaxial surface brown to dark green when dry, densely to moderately stellate-pubescent and prickly, the trichomes like those of the stem but with (1–)4–6 rays, the prickles along the midrib and major veins, to 5.5 mm long and 1 mm wide at the base, straight and laterally compressed; abaxial surface whitish-green when dry, more densely stellate-pubescent than the adaxial surface, the trichomes like those of the adaxial surface, the prickles like those of the adaxial surface but to 6.5 mm long and 2 mm wide at the base; base attenuate to truncate or rounded, less often with 1 or 2 basiscopic lobes, decurrent onto the petiole, sometimes asymmetrical; margins shallowly lobed, the lobes (0)3–5 on each side, 1–12(14.8) mm long, 3.2–11(23) mm wide at base with usually acute, sometimes rounded or obtuse apices, the sinuses 3.2–8.5 mm deep; apex acute to acuminate; primary veins 4–6 pairs, more prominent beneath, prickly on both surfaces; petioles 0.6–3.3 cm long, densely to moderately pubescent with porrect-stellate trichomes like those of the leaves, usually armed with 1–5 prickles. Inflorescence a monochasial cyme (drepanium) to 6 cm long, internodal, unbranched, with 4–10 flowers, up to 2 flowers open at a time, the axes glabrescent to densely tomentose, with trichomes like those of the stem but sometimes with the midpoint as long as the rays, usually unarmed; peduncles 0.4–2.3 (-3.8) cm long pedicels 3–17 mm long, 0.5–0.8 mm in diameter at base and up to 1.5 mm at the apex, straight to slightly curved, articulated at base, unarmed, with trichomes like those of the inflorescence axes; pedicel scars evenly spaced 1–7 mm apart. Buds ovoid to ellipsoid, with the corolla enclosed by the calyx until just before anthesis. Flowers 5-merous, heterostylous with long-styled flowers (hermaphroditic) at the base of inflorescence, short-styled (functionally male) flowers more distally and the plants andromonoecious. Calyx tube 2.6–4.3(6) mm long, 6.5–8 mm in diameter at anthesis, widely obconic to cupuliform, the lobes 3–7 mm long, 3–5.5 mm wide, triangular to deltate, with acute apices, glabrous adaxially, densely pubescent to hirsute abaxially with bristly purple-tinged, hyaline or ochraceo-ferruginous porrect to multiangulate long-stalked stellate trichomes, the stalks of the fully developed trichomes multiseriate, 1.1–3.8 mm long, rays 4-8 to 1.5 mm, the midpoints 1–2 celled, shorter than or the same length as the rays, associated with minute, nearly sessile, uniseriate, simple glandular trichomes along the epidermis and sometimes at the basal portion of the stellate trichomes stalks, armed or unarmed, the prickles acicular, 2.8-4 mm long, ca. 0.5 mm wide in flower to 1.1 mm in fruit. Corolla 2.4–3 cm in diameter, 7.1–12.2 mm long, white, shallowly stellate, interpetalar tissue nearly absent, lobed ca. halfway to the base, the fused part (tube) 7.1–12.2 mm long, the lobes 5.9–8.8 mm long, 9.9–12.2 mm wide, acute to apiculate apices, pubescent abaxially on the petal midvein and/or apices with sparse delicate short-stalked porrect-stellate trichomes with stalks to 0.9 mm long. Stamens equal; filament tube to 1 mm long; free portion of the filaments 0.7–1 mm long, glabrous; anthers 6.5–8 mm long, 2.5–3 mm wide, lanceolate, yellow, glabrous, connivent or divergent at the apices, sagittate and slighty gibbous at the base, swollen and papillose abaxially, the pores directed upwards or slightly extrorse, not lengthening to slits. Ovary somewhat conical, glabrous; style 8–10 mm long in long-styled flowers, ca. 3 mm long in short-styled flowers, straight, glabrous; stigma clavate to bilobed, the surface papillose and irregular, the style poorly developed in short-styled flowers. Fruit a globose berry, 0.9–1.8 cm in diameter, green to whitish-green at maturity, drying dark brown, glabrous, matte; fruiting pedicels 1–1.5 cm long, usually unarmed; fruiting calyx partially accrescent, the tube enclosing ½–¾ of the fruit at maturity, the lobes 5.8–8 mm long, 7–9.6 mm wide, with trichomes often with the base of the stalks markedly expanded and bristly, the stalks to 4.8 mm long. Seeds ca. 250 per berry, 2.2–2.6 mm long, 1.6–2 mm wide, pyriform to reniform, not markedly flattened, the surface irregularly pitted, the testal cells pentagonal in outline. Chromosome number not known.

**Figure 1.**
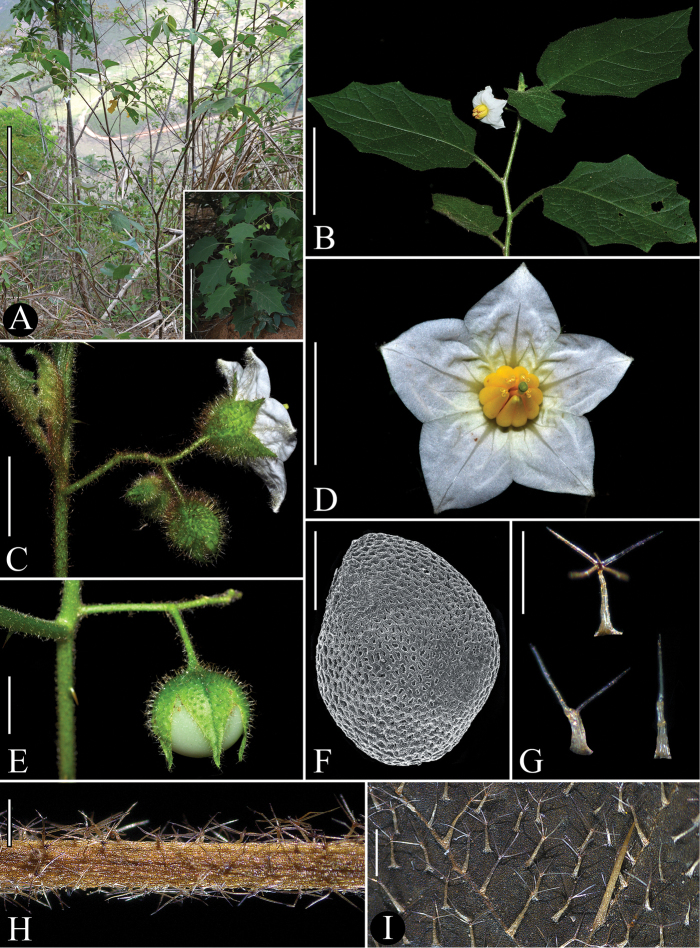
*Solanum hydroides* Gouvêa & Giacomin. (**A**, **G**–**I** field pictures from specimens *L.F.A. de Paula et al. 669*, BHCB; **B–E***Y.F. Gouvêa & G.V.A. Santos 325*, BHCB). **A** Habit (bottom right corner: young plant with larger leaves) **B** flowering branch **C** inflorescence and a flower in lateral view (note that calyx does not have a plicate aspect at the base of the calyx tube) **D** long-styled flower, front view **E** mature fruit (note the calyx does not completely cover the berry) **F** scanning electron micrograph of seed **G** trichomes; upper: the usual morphology of the stellate trichomes of *S. hydroides* adaxial leaf surface; lower: examples of stellate trichomes with reduced number of rays (note the multiseriate stalks) **H** stem indumentum; **I** adaxial leaf surface indumentum. Scale bars: 30 cm (**A**); 7.5 cm (**B**); 1.3 cm (**C**–**D**); 1 cm (**E**); 0.8 mm (**F**–**I**). Photographs: **A** by L.F.A de Paula **B**–**E**, **G**–**I** by Y.F. Gouvêa.

#### Distribution.

**Figure 2.**
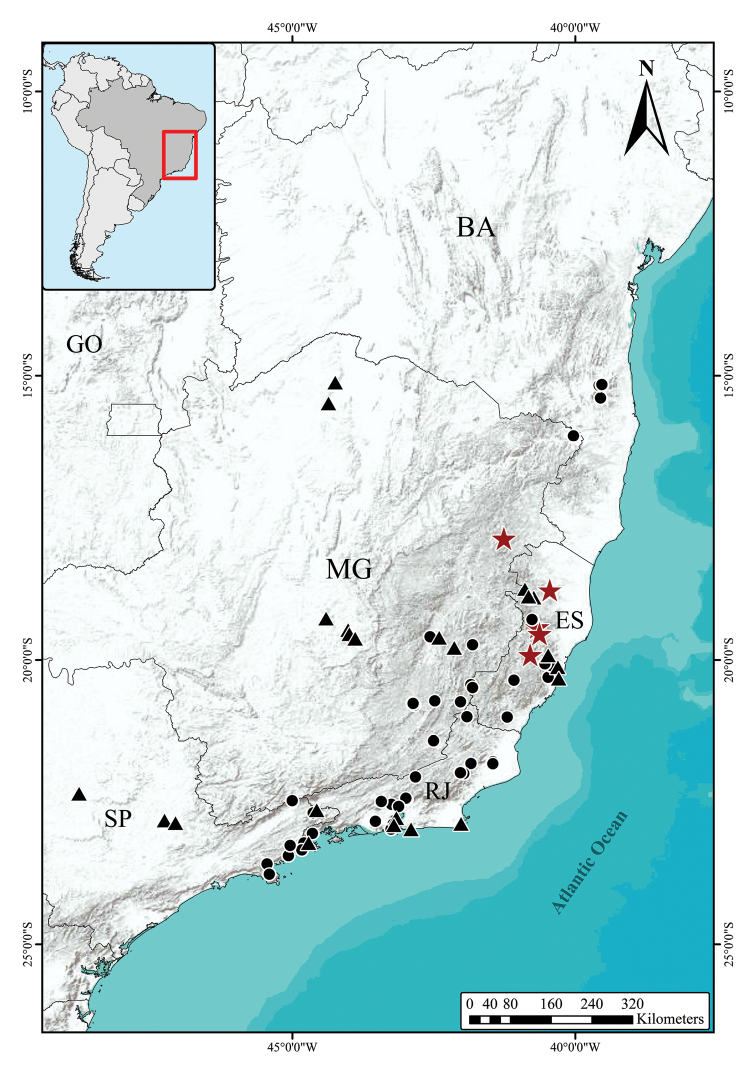
Distribution of *Solanum hydroides* Gouvêa & Giacomin (stars), *S. hexandrum* Vell. (circles) and *S. sublentum* Hiern. (triangles). State acronyms: **BA** (Bahia); **ES** (Espírito Santo); **MG** (Minas Gerais); **GO** (Goiás); **RJ** (Rio de Janeiro); **SP** (São Paulo).

Endemic to South-eastern Brazil, with records in three localities in north-eastern Minas Gerais (Mun. Teófilo Otoni) and northern (Mun. Nova Venécia) and central (Mun. Santa Teresa) Espírito Santo States (Fig. 2).

#### Ecology.

*Solanum hydroides* inhabits the edge of seasonal semi-deciduous tropical rainforests associated with granitic or gneissic rock outcrops (inselbergs) and somewhat disturbed sites at their base, like roadsides and clearings; from 300 to 600 m elevation. It also occasionally grows in epilithic vegetation patches lying on the flatter parts of inselbergs.

#### Phenology.

Flowering specimens were collected in January, April, May, August, September and December, suggesting that *S. hydroides* blooms year-round. Fruiting specimens have been found only in January.

#### Etymology.

*Solanum hydroides* is named for the resemblance of the long-stalked stellate trichomes of its calyces to the marine serpulid worm *Hydroides* Gunnerus, 1768 (illustrative images can be found at the Encyclopedia of Life; e.g. https://eol.org/search?utf8=%E2%9C%93&q=Hydroides).

#### Preliminary conservation status (IUCN 2019).

Vulnerable (VU). EOO = 7,766 km^2^ (VU - vulnerable); AOO = 28 km^2^ (EN - endangered). *Solanum hydroides* is known from only three disjunct localities and is represented by only one collection in one of them (i.e. Serra do Toma Vento, Mun. Santa Teresa, Espírito Santo State). All other specimens were found in vegetation remnants associated with two inselbergs, located in the municipality of Nova Venécia, Espírito Santo state and in Minas Gerais State’s municipality of Teófilo Otoni. Both, however, are in the central Brazilian Atlantic Forest, where botanical knowledge gaps are known to exist (Stehmann et al. 2009; Oliveira et al. 2016), suggesting the actual range of *S. hydroides* might be broader than currently known. Further sampling efforts in environmentally similar areas are therefore recommended, for a more accurate conservation status assessment. Nonetheless, the deforestation history of the area and the available geographic distribution data indicate that *S. hydroides* is of conservation concern.

#### Notes.

*Solanum hydroides* shares a set of morphological features with species of a small and still unnamed group, recently proposed on the basis of morphological (Gouvêa et al. 2018) and molecular evidence (Giacomin et al., unpubl. data). This group, endemic to the Brazilian Atlantic Forest, comprises five previously described species: *S. hexandrum*, *S. kollastrum* Gouvêa & Giacomin, *S. robustum* H.Wendl., *S. stagnale* Moric. and *S. sublentum*. They are all medium- to large-sized shrubs with large repand leaves with decurrent bases (except *S. kollastrum*, which has cordate leaf bases), relatively robust and showy flowers with well-developed calyces that are accrescent in fruit and glabrous fruits (except *S. robustum*, which has weakly accrescent fruiting calyces and densely pubescent fruits).

**Figure 3.**
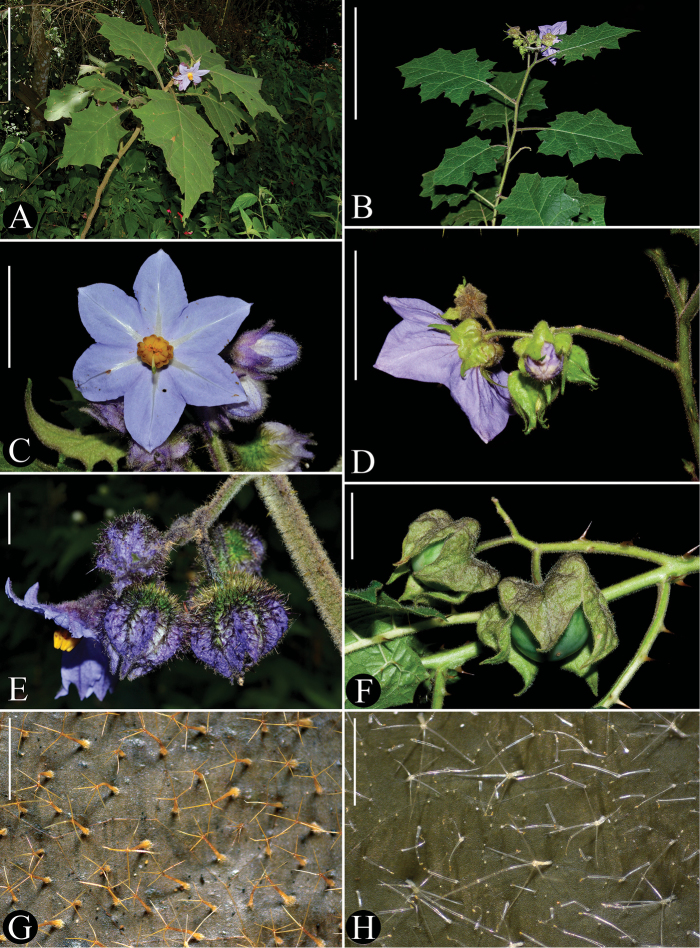
Distinctive characters of species morphologically related to *Solanum hydroides* Gouvêa & Giacomin. [**A**, **C**, **E**, **G**: *S. hexandrum* Vell. (*L.L. Giacomin et al. 875*, BHCB); B, D, F, H: *S. sublentum* Hiern. (*J.R. Stehmann et al. 6372*, BHCB)]. **A–B** Habit (note difference in robustness) **C** long-styled flower, front view **D** inflorescence and flower in back view (note the inflated and plicate aspect of the calyx tube) **E** inflorescence with fruits in different stages of development (note that the inflated fruiting calyx completely covers the fruit in all stages of development) **F** long-styled flower **G** fruits (note the plicate aspect of the fruiting calyx) **H**–**I** indumentum of the adaxial leaf surface. Scale bars: 15 cm (**A**); 10 cm (**B**); 3 cm (**C**); 2 cm (**D**, **G**); 1.4 cm (**E**); 1 mm (**F**); 1.2 mm (**H**). Photographs: **A**, **C**, **E** by L.L. Giacomin **B**, **D**, **F** by J.R. Stehmann **G**–**H** by Y.F. Gouvêa.

The comparatively smaller leaves and thinner stems, petioles and inflorescence axis give a more delicate overall aspect of *S. hydroides*, differentiating it from all other species of the group, but *S. sublentum*. *Solanum hydroides* can, however, be readily distinguished from *S. sublentum* by the indumentum of stellate eglandular trichomes (Figs 1G–I) and by the widely obconic to cupuliform shape of the calyx at anthesis (Fig. 1C). In *S. sublentum*, the indumentum is of both conspicuous simple glandular trichomes and stellate eglandular trichomes (Fig. 3H), with the stellate trichomes usually much less numerous than the simple ones and often early deciduous (i.e. present only in new growth). Calyces of *S. sublentum* are somewhat urceolate, inflated and prominently plicate at the base of the calyx tube (Fig. 3D).

Although being a markedly less robust species (compare Figs 1A and B with 3A), *S. hydroides* can be very similar to some specimens of *S. hexandrum* (a quite variable species), with which it shares the indumentum of four-rayed stellate eglandular trichomes (Figs 1H, I and 3G) on the stems, leaves, inflorescence axis and calyces. *Solanum hydroides* differs from *S. hexandrum* in its white and smaller corollas (13–21.5 mm total length), shorter corolla lobes (5.9–8.8 mm long; Fig. 1D), and accrescent, but not inflated, fruiting calyces that only partially cover the mature fruit (Fig. 1E). *Solanum hexandrum* has corollas in various shades of lilac to purple and are larger (24.3–40 mm long), with longer corolla lobes (12.6–25 mm long; Fig. 3C) and the fruiting calyces are accrescent and inflated, completely enclosing the mature fruit (Fig. 3E). The corollas of *S. hydroides* are thin and membranous and easily tear apart between the lobes during the drying process, which can make the lobes on herbarium specimens seem longer than they really are. Hence, one should carefully check before measuring to ensure a correct measurement is taken from herbarium sheets.

Leaf measurements are also useful for distinguishing *S. hydroides* from *S. hexandrum*. The leaves of *S. hydroides* are generally smaller (7.5–13.6 cm long and 5-8.7 cm wide) than those of *S. hexandrum* (17–45 cm long and 10.5–32 cm wide). Nevertheless, like many species belonging to the Leptostemonum clade (sensu Bohs 2005; Stern et al. 2011), the leaves of *S. hydroides* are larger in plants growing in shade and in young individuals (see Roe 1966) and we have seen plants with leaves to 22 cm long and 11 cm wide. Specimens of *S. hydroides*, growing in shade, have a less dense indumentum, with less robust (i.e. stalks with fewer series of cells) and slightly shorter trichomes on stems, leaves and calyx. Corollas of these shade plants are usually larger in relation to the other flower parts (e.g. stamens and calyx).

The trichome morphology in *S. hydroides* is not particularly variable within individual plants and amongst plants of the same population; however, there is a significant variation in the number of trichome rays between some populations. Trichomes of specimens from the southernmost known population (in Santa Teresa municipality, Espirito Santo State) are mostly six- to eight-rayed and usually more densely distributed throughout the plant, while those of plants from the other populations (Teófilo Otoni and Nova Venécia municipalities of Minas Gerais State) are mostly four-rayed (Figs 1H and I). Within individual plants, the variation in trichome morphology is limited to a reduction in the number of rays and is especially evident in plants with four-rayed trichomes. In these plants, the trichomes may lack one to almost all rays (Fig. 1G), sometimes with only the midpoint or a lateral ray remaining and the trichome appearing to be unbranched, but with a basal multiseriate stalk. This kind of variation has been reported in other *Solanum* groups, such as the Brevantherum clade or members of section Acanthophora Dunal (Nee 1991; Levin et al. 2005; Stern et al. 2013).

The exploitation of natural resources in Brazil is far from being sustainable (Ferreira et al. 2014) and this fact, combined with the rapid fragmentation of the Atlantic Forest (Tabarelli et al. 2004), raises the risks for the vegetation refugia associated with inselbergs found in the domain. Particularly in some regions in south-eastern Brazil, these rock outcrops harbour the last remnants of forest fragments (Martinelli 2007). Therefore, we argue that every remnant of native vegetation of rocky outcrops, no matter the size, is worth preserving and should be inspected. Remnants can harbour new species and endemics, like *S. hydroides*, despite the massive fragmentation and loss of the surrounding vegetation.

#### Specimens examined.

**BRAZIL**. **Espírito Santo**: Mun. Nova Venecia, APA da Pedra do Elefante, Serra de Baixo, Pedra do Elefante, inselbergue, 18°46'S, 40°27'W, 653 m elev., 10 May 2008 (fl), *A.P. Fontana et al. 5259* (MBML, RB); Serra de Baixo, Pedra da Torre, inselbergue, 18°46'58"S, 40°26'47"W, 420–500 m elev., 18 Feb 2008 (fr), *C.N. Fraga et al. 1899* (CEPEC, MBML, RB, UPCB); estrada não pavimentada de acesso à Pedra do Elefante, 18°46'40"S, 40°26'37"W, 352 m elev., 1 Apr 2019 (fl, fr), *Y.F. Gouvêa & G.V.A. Santos 325* (BHCB); 18°46'42"S, 40°26'50"W, 301 m elev., 1 Apr 2019 (fl, fr), *Y.F. Gouvêa & G.V.A. Santos 328* (BHCB); morro lado direito na estrada para Pedra do Elefante, afloramento rochoso, inselbergue, 18°46'12"S, 40°26'51"W, 300–600 m elev., 14 Jan 2009 (fl), *L. Kollmann et al. 11385* (CEPEC, MBML, RB, UPCB); Mun. Santa Teresa, Serra do Toma Vento, em inselberg, 19°54'29"S, 40°47'44"W, 747 m elev., 26 Aug 2014 (fl), *T.M. Machado et al. 673* (BHCB). **Minas Gerais**: Mun. Teófilo Otoni, afloramento rochoso lado esquerdo da MG-418, cerca de 30 km norte de Teófilo Otoni, 17°51'33"S, 41°15'46"W, 546 m elev., 8 Jan 2011 (fl, fr), *L.F.A. de Paula 148 et al.* (BHCB); 16 Apr 2011 (fl), *L.F.A. de Paula* & *M. Augsten 247* (BHCB); 17°51'42.1"S, 41°15'54.4"W, 533 m elev., 9 Sep 2011 (fl), *L.F.A. de Paula et al. 388* (BHCB); 17°51'45.9"S, 41°16'00.5"W, 450 m elev., 27 Dec 2011 (fl), *L.F.A. de Paula et al. 581* (BHCB).

## Supplementary Material

XML Treatment for Solanum hydroides

## Supplementary materials

Supplementary material 1 Searchable XLS file of all specimens examined of *Solanum hydroides* Gouvêa & Giacomin Data type: species data https://binary.pensoft.net/file/375529This dataset is made available under the Open Database License (http://opendatacommons.org/licenses/odbl/1.0/). The Open Database License (ODbL) is a license agreement intended to allow users to freely share, modify, and use this Dataset while maintaining this same freedom for others, provided that the original source and author(s) are credited.Yuri Fernandes Gouvêa, Luiza Fonseca Amorim de Paula, João Renato Stehmann, Leandro Lacerda Giacomin

Supplementary material 2 Searchable XLS file of all records used to map distributions of *Solanum hexandrum* Vell. and *Solanum sublentum* Hiern Data type: species data https://binary.pensoft.net/file/375530This dataset is made available under the Open Database License (http://opendatacommons.org/licenses/odbl/1.0/). The Open Database License (ODbL) is a license agreement intended to allow users to freely share, modify, and use this Dataset while maintaining this same freedom for others, provided that the original source and author(s) are credited.Yuri Fernandes Gouvêa, Luiza Fonseca Amorim de Paula, João Renato Stehmann, Leandro Lacerda Giacomin
